# Heat Shock Protein 90 as a Prognostic Marker and Therapeutic Target for Adrenocortical Carcinoma

**DOI:** 10.3389/fendo.2019.00487

**Published:** 2019-07-19

**Authors:** Claudia Siebert, Denis Ciato, Masanori Murakami, Ludwig Frei-Stuber, Luis Gustavo Perez-Rivas, José Luis Monteserin-Garcia, Svenja Nölting, Julian Maurer, Annette Feuchtinger, Axel K. Walch, Harm R. Haak, Jérôme Bertherat, Massimo Mannelli, Martin Fassnacht, Esther Korpershoek, Martin Reincke, Günter K. Stalla, Constanze Hantel, Felix Beuschlein

**Affiliations:** ^1^Medizinische Klinik und Poliklinik IV, Klinikum der Universität, Ludwig-Maximilians-Universität München, Munich, Germany; ^2^Department of Clinical Endocrinology, Max Planck Institute of Psychiatry, Munich, Germany; ^3^Research Unit Analytical Pathology, Helmholtz Zentrum München, German Research Center for Environmental Health, Neuherberg, Germany; ^4^Department of Internal Medicine, Máxima Medical Center, Eindhoven, Netherlands; ^5^CAPHRI School for Public Health and Primary Care, Ageing and Long-Term Care, Maastricht University, Maastricht, Netherlands; ^6^Division of General Internal Medicine, Department of Internal Medicine, Maastricht University Medical Centre, Maastricht, Netherlands; ^7^Department of Endocrinology, Hôpital Cochin, Paris, France; ^8^Endocrine Unit, Department of Clinical Pathophysiology, University of Florence, Florence, Italy; ^9^Division of Endocrinology and Diabetes and Central Laboratory, Department of Internal Medicine I, University Hospital, University of Würzburg, Würzburg, Germany; ^10^Department of Internal Medicine, Erasmus MC, Rotterdam, Netherlands; ^11^Medicover Neuroendocrinology, Munich, Germany; ^12^Klinik für Endokrinologie, Diabetologie und Klinische Ernährung, UniversitätsSpital Zürich, Zurich, Switzerland; ^13^Endokrinologie, Medizinische Klinik und Poliklinik III, Universitätsklinikum Carl Gustav Carus, Dresden, Germany

**Keywords:** adrenal gland, cortisol, N-terminal HSP90 inhibitors, C-terminal HSP90 inhibitors, prognostic marker

## Abstract

**Background:** Adrenocortical carcinoma (ACC) is a rare tumor entity with restricted therapeutic opportunities. HSP90 (Heat Shock Protein 90) chaperone activity is fundamental for cell survival and contributes to different oncogenic signaling pathways. Indeed, agents targeting HSP90 function have shown therapeutic efficacy in several cancer types. We have examined the expression of HSP90 in different adrenal tumors and evaluated the use of HSP90 inhibitors *in vitro* as possible therapy for ACC.

**Methods:** Immunohistochemical expression of HSP90 isoforms was investigated in different adrenocortical tumors and associated with clinical features. Additionally, a panel of N-terminal (17-allylamino-17-demethoxygeldanamycin (17-AAG), luminespib, and ganetespib) and C-terminal (novobiocin and silibinin) HSP90 inhibitors were tested on various ACC cell lines.

**Results:** Within adrenocortical tumors, ACC samples exhibited the highest expression of HSP90β. Within a cohort of ACC patients, HSP90β expression levels were inversely correlated with recurrence-free and overall survival. In functional assays, among five different compounds tested luminespib and ganetespib induced a significant decrease in cell viability in single as well as in combined treatments with compounds of the clinically used EDP-M scheme (etoposide, doxorubicin, cisplatin, mitotane). Inhibition of cell viability correlated furthermore with a decrease in proliferation, in cell migration and an increase in apoptosis. Moreover, analysis of cancer pathways indicated a modulation of the ERK1/2—and AKT—pathways by luminespib and ganetespib treatment.

**Conclusions:** Our findings emphasize HSP90 as a marker with prognostic impact and promising target with N-terminal HSP90 inhibitors as drugs with potential therapeutic efficacy toward ACC.

## Introduction

Adrenocortical carcinoma (ACC) represents a rare tumor entity with variable but overall poor clinical outcome (1, 2). Morbidity and mortality are defined by local space occupying and infiltrating effects of the primary tumor, by metastatic spread to distant organs and by the excessive secretion of steroid hormones that result in Cushing's syndrome and/or hyperandrogenism. Despite recent progress in the understanding of molecular mechanisms contributing to tumor growth and the identification of genomic markers with prognostic value (3, 4), it has not yet been translated into novel therapeutic approaches. In fact, clinical studies evaluating targeted molecular therapies have been mostly disappointing, including one placebo controlled trial on inhibition of IGF2 dependent signaling (5). Further targets that have been identified as affected by point mutations, chromosomal alterations or epigenetic mechanisms including p53/Rb or the Wnt/β-catenin dependent signaling still lack suitable pharmacological approaches. As a result, medical treatment of ACC patients not amendable to surgical resection has barely changed over the last decade (6). Current standard therapeutic regiments include doxorubicin, etoposide and cisplatin (EDP) in combination with the adrenolytic substance mitotane (7). However, the protocol carries a high burden of toxic side effects and remains to be associated with poor overall survival.

Heat shock proteins (HSPs) comprise a family of evolutionary conserved molecules that are essential to protect cells against stressor events and during growth conditions (8). HSPs generally exert chaperoning functions, stabilizing unfolded precursor proteins or the resolution of protein aggregates by direct binding and ATP-dependent mechanisms (8).

Among these, HSP90 has emerged as a pivotal factor for the survival of cancer cells. The protein is mostly located in the cytoplasm, with about 5–10% localized in the nucleus. While its cytoplasmic role is mainly ascribed to stabilization of client proteins, its nuclear fraction was described to drive nuclear transport of proteins like hormone receptors and protein kinases (9, 10). There are two major isoforms of HSP90 in vertebrates, HSP90α, and HSP90β, whether minor forms, such as HSP90 N and a mitochondrial isoform are reported (9, 11, 12). HSP90 is mainly formed by constitutive homodimers dimers, but monomers (especially for HSP90β) or heterodimers and higher oligomers of both isoforms may also be present. HSP90α is described as strongly inducible by sources of cellular stress such as heat shock and overexpressed in cancerous cells, whether the HSP90β is constitutively expressed in most tissues and much lesser inducible (12–14). Unlike the difficult separation of the two isoforms and the absence of differentiation between HSP90α and HSP90β in most functional studies (12), considerable evidences were reported about their respective biochemical function. Treatment of Zebrafish embryos with the N-terminal HSP90 inhibitor Geldanamycin exhibited differential effects in tissues expressing one or the other gene during muscle differentiation (15). Yamada et al. described a possible differential impact of HSP90α or β isoform overexpression in the differentiation of human embryonal carcinoma cells (16). HSP90β was shown to potentially play a role in different stages of embryonic development (13, 17–19) and was shown to bind to tubulin and actin with higher frequency than HSP90α and other heat shock proteins, suggesting a role in cytoskeleton structure organization (20). The general structure of the protein homodimer is composed by a highly conserved amino-terminal domain (NTD) connected to a middle domain (MD), followed by a carboxy-terminal domain (CTD). The overall structure of the NTD is an α/β sandwich with a pocket for a nucleotide-binding site. The structures of the NTDs with nucleotide bound confirmed that HSP90 is an ATP-binding protein with ATPase activity (21). This evidence was confirmed by subsequent mutagenesis studies and studies of HSP90 crystal structure with natural HSP90 inhibitors proved their nucleotide-mimicry effect for inhibition of ATPase activity (22, 23).

The structure of MD comprises two structural subdomains—a large α/β/α domain linked to a smaller α/β/α domain (21, 24). Although the catalytic center of HSP90 is in the NTD of the protein, some evidences suggested an additional role by MD of the protein to regulate bind, chaperoning, and release of client proteins (25, 26).

The CTD is a homodimer of two small mixed α/β domains, where the dimer interface is formed by two α-helices which pack together to form a four-helical bundle (27, 28). This domain plays a fundamental role in the conformational change of HSP90 to favor its dimerization (29). Analysis of a series of truncation mutants of HSP90 showed that different regions at the CTD were involved in trapping the bound ATP to maximize hydrolysis rates leading to a repositioning of both NTD and CTD (30). HSP NTD and CTD are the principal targets of specific inhibitors that were discovered or synthetized over the last two decades.

The breakthrough for the druggability of HSP90 was found by the discovery of the target of the natural compounds geldanamycin and radicicol (31, 32). Both drugs act by selective inhibition of ATP binding and hydrolysis, with the consequent degradation of oncogenic clients (33–35). Subsequent improvements of these molecules—such as the better-tolerated geldanamycin analog 17-allylamino-17-demethoxygeldanamycin (tanespimycin, 17-AAG)—could reach clinical-trial use but showed limited solubility and liver toxicity (36, 37). HSP90 CTD has also been investigated for its suitability to be targeted by pharmacological agents. Coumarin antibiotics, such as novobiocin, and an extract from milk thistle seeds named silibinin showed the ability to induce apoptosis in cancer cells, in some cases with superior efficacy compared with tanespimycin (38). Noteworthy, silibinin treatment led to a release of mature glucocorticoid receptor (GR) from the chaperoning complex, partially restoring glucocorticoid sensitivity in an allograft model for Cushing's disease (39). Subsequent efforts testing diverse chemical scaffolds led to the development of highly potent, second-generation, small molecule HSP90 inhibitors with improved pharmacological properties and safety profiles. This is the case of the N-terminal inhibitors luminespib and ganetespib, which lack the hepatoxicity noted for the first-generation inhibitors due to the absence of the benzoquinone moiety in their structure (40, 41). Both compounds showed potent preclinical efficacy in a wide range of tumor types for instance in lung and prostate cancer and reached phase II and phase III studies, respectively^1^ (42, 43).

In the current study, we make usage of a large patient collection to evaluate the expression of HSP90 as a potential prognostic marker and therapeutic target. We further aimed to translate these clinical data in suitable *in vitro* models to substantiate initial findings and to identify underlying molecular mechanism that follows targeting of specific HSP90 domains. Thereby, we present evidence that specific N-terminal HSP90 inhibitors could provide promising treatment opportunities for ACC patients that could be tested in future clinical studies.

## Materials and Methods

### Patient Cohorts

Two groups of patients with adrenocortical tumors were included in the immunohistochemical analysis. Patient group 1 consisted of 32 patients with adrenal tumors: eight patients with nonfunctional adenomas (NFA), 18 patients with cortisol-secreting adenomas [four patients with autonomous cortisol secretion without signs of overt Cushing (ACS) and 14 patients with overt Cushing's syndrome (CS)] and six patients with adrenocortical carcinoma (ACC). All of those were diagnosed and surgically treated at one referral center (Klinikum der Ludwig-Maximilians-Universität München, Munich, Germany). Patient group 2 was comprised of a cohort of 80 ACC patients from six European centers. Diagnostic work-up was done following established criteria based on clinical, biochemical, and morphological data according to recent ESE/ENSAT guidelines (44, 45). Clinical data were collected through the ENSAT database^2^. All patients had provided written informed consent and the study was approved by ethics committees at all participating institutions (Medizinische Fakultät der Universität München, Maastrich University, Hôpital Cochin Paris, University of Florence, and Universität Würzburg).

### Immunohistochemistry and Quantification

Archival tumor blocks from patient group 1 were sectioned following standard procedures. For patient group 2, tissue microarrays were constructed by sampling three tumor tissue cores (1.0 mm in diameter) from each paraffin-embedded tissue block, which were selected based on representative hematoxylin-eosin stained tissue sections. The construction of the tissue microarrays was performed on an automated TMA constructor (ATA-27; Beecher Instruments, Sun Prairie, WI) available at the Department of Pathology, Erasmus MC, as previously described (46). Tumor samples were deparaffinized and microwaved in 100 mM Tris-HCl pH 8.0, 10 mM EDTA for antigen retrieval. Non-specific background was blocked by incubation with 0.03 v/v H_2_O_2_ in methanol for 10 min. and with 0.2 v/v human AB serum (Merck, Darmstadt, Germany) in 100 mM Tris-HCl pH 7.6, 0.001 v/v Tween® 20 for 1 h at RT. The proteins of interests were detected using primary antibodies specifically directed against human HSP90α/β (1:300 in blocking buffer, clone ERP3953, Abcam, Cambridge, UK), HSP90β (1:750, E296, Abcam, Cambridge, UK) and stained using the EnVision Detection System (DAKO-Kit, Santa Clara, United States). For further immunohistochemical studies, tumor blocks from xenografted MUC-1 and NCI-H295R cells were utilized. For NCI-H295R (47), 15 × 10^6^ cells in a volume of 200 μl PBS had been inoculated into female athymic NMRI *nu/nu* mice, while for MUC-1 (48), the originally established xenograft from patient material had been repeatedly passed over into other groups of animals. Immunohistochemical staining of NCI-H295R and MUC-1 xenograft tissues was performed as described above excepted antigen retrieval was conducted with 10 mM citrate buffer pH = 6.0 for HSP90α/β and non-specific background was blocked with 0.01 v/v H_2_O_2_ in methanol. Xenograft tissues were counter-stained with Harris Hematoxylin (Sigma-Aldrich). Slides were dehydrated and mounted with Roti®-Histokitt II (Roth, Karlsruhe, Germany) and pictures were taken by optical microscope Leica DMRB and a Leica DMC 2900 digital camera (Leica, Wetzlar, Germany).

For patient group 1, levels of HSP90α/β and HSP90β were evaluated by means of a semi-quantitative H-score with four categories (0 no immunoreactivity, 1 low immunoreactivity, 2 intermediate immunoreactivity, and 3 high immunoreactivity) followed by calculation of individual scores for each high power field image using the formula H = n0^*^0+n1^*^1+n2^*^2+n3^*^3/(3^*^n), where n refers to the number of counted cells and calculation of the average H-score for 10 representative high power fields. For patient group 2, stained tissue microarray slides were scanned at ×20 objective magnification using a digital Mirax Desk slide scanner (Carl Zeiss Microscopy GmbH, Jena, Germany) and analyzed with the image analysis software Definiens Developer XD2 (Definiens AG, Munich, Germany), as previously described (49). Tumor areas were annotated manually, and a setup of rules was defined to detect and quantify staining intensities in the annotated tumor area, with operators blinded with regard to corresponding *in vivo* steroid output.

### Cell Culture and Primary Culture

Two human adrenocortical carcinoma cell lines were used for these experiments, NCI-H295R cells (ATCC) and the newly developed MUC-1 cell line (48). NCI-H295R cells were cultured in RPMI (1640) plus GlutaMAX (Thermo Fisher Scientific Gibco, Waltham, MA, USA) supplemented with 0.1 v/v fetal bovine serum (FBS, Thermo Fisher Scientific Gibco), 0.01 v/v, penicillin/streptomycin (Thermo Fisher Scientific Gibco) and 0.01 v/v insulin-transferrin-selenium (ITS, Thermo Fisher Scientific Gibco). The culture medium of MUC-1 was advanced DMEM/F12 (Thermo Fisher Scientific Gibco), containing 0.1 v/v FBS and 0.01 v/v penicillin/streptomycin. The treatment medium of MUC-1 cells was DMEM/F12 (Thermo Fisher Scientific Gibco) containing 0.02 v/v charcoal stripped serum (Thermo Fisher Scientific Gibco) and 0.01 v/v penicillin/streptomycin. For NCI-H295R further 0.01 v/v ITS and 0.01 v/v glutamine were added.

For primary culture, we utilized samples of metastases from a 32-year-old female with a history of an androgen and cortisol producing ACC. Upon pathological examination, surgical tumor samples were cleaned from fat and connective tissues followed by separation in small pieces and centrifugation at 1100 rpm for 5 min. Upon removal of collagen and erythrocytes, cells were separated from larger tumor pieces. Finally, cells were seeded in RPMI (1640) plus GlutaMAX, 0.1 v/v fetal calf serum (FCS, Biochrom KG, Berlin, Germany), 0.01 v/v penicillin/streptomycin and 0.004 v/v amphotericin B (Biochrom KG).

### Immunofluorescence

NCI-H295R (1.5 × 10^5^ to 1.8 × 10^5^) and MUC-1 (8 × 10^4^ to 1 × 10^5^) cells were seeded in 4-well chamber slides (Sarstedt, Nümbrecht, Germany) and incubated overnight. Afterwards, cells were washed twice with PBS and fixed with 1 mL/well with freshly prepared 0.04 v/v paraformaldehyde solution for 30 min. at RT. Following two washing steps with PBS, immunofluorescence staining for HSP90α/β and HSP90β was conducted according to the protocol for immunohistochemistry except for the dehydration/rehydration steps and inhibition of endogenous peroxidases. Alexa Fluor®594 donkey-anti rabbit (1:200, Cell Signaling, Cambridge, United Kingdom) was used as secondary AB and slides were covered with Vectashild mounting medium with DAPI (Vector Laboratories Inc. Burlingame, USA).

MUC-1 cells were stained with monoclonal antibodies specific for ERK1/2 (1:100, Cell Signaling) and p-ERK1/2 (1:250, Cell Signaling). The secondary AB Alexa Fluor®488 donkey-anti rabbit (1:200, Cell Signaling) was used and slides were mounted as described before. Staining was documented using the fluorescence microscope Leica DM2500 with a Leica DFC 340 FX camera (Leica, Wetzlar, Germany).

### Cell Viability

MTT assays were performed in a 96-well plate format. NCI-H295R (5 × 10^3^ per well) and MUC-1 (6 × 10^3^ per well) were seeded in 100 μL culture medium for single HSP90 inhibitor treatment in variable concentrations with ganetespib, luminespib (both from Selleckchem, Houston, TX, USA), 17-AAG (Tocris, Bristol, UK), silibinin (Sigma-Aldrich), and novobiocin (Calbiochem, San Diego, United States), respectively. For combined treatment (HSP90 inhibitor + compounds from the EDP-M scheme) 8 × 10^3^ NCI-H295R cells/ well and 6 × 10^3^ MUC-1 cells/ well were seeded in 100 μL culture medium. Following the individual incubation durations, medium was removed and 0.5 mg/ mL MTT (Sigma-Aldrich) (100 μL/well) was added to the cells for 2 h. The reaction was stopped by adding 0.1 v/v SDS (100 μL/ well). Absorbance at λ = 570 nm and λ = 655 nm was measured in a TECAN Sunrise™ absorbance reader (Männedorf, Switzerland) and an iMark™ microplate absorbance reader (Bio-Rad Laboratories Inc., Hercules, United States).

Celltiter Blue assay (Promega, Mannheim, Germany) was performed in 96-well plate format for primary culture cells. ACC cells (2 × 103 per well, 100 μl/well) were seeded and treated with 0.2 μM luminespib and 0.2 μM ganetespib for 72 h. Afterwards Celltiter Blue assay was conducted following the manufacturer's instructions.

### Cell Proliferation ELISA 5-bromo-2′-deoxyuridine (BrdU)

NCI-H295R (8 × 103 cells per well) and MUC-1 (6 × 103 cells perwell) cells were treated with HSP90 inhibitors (luminespib, ganetespib, 17-AAG, novobiocin and silibinin) for 12 and 48 h, respectively. BrdU assay was performed using cell proliferation ELISA (BrdU, colorimetric immunoassay) (Roche, Basel, Switzerland) for quantification following manufacturer's instructions.

### Apoptosis (Caspase-3/7 Assay)

NCI-H295R (8 × 103 cells per well) and MUC-1 (8 × 103 cells per well) cells were seeded on 96-well-plates and incubated overnight following incubation with the respective HSP90 inhibitors for 24 h. For quantification of caspase-3 and caspase-7 activity the luminescent assay Caspase-Glo 3/7 (Promega, Madison, Wisconsin, United State) was used following the manufacture's protocol with luminescence detection in a Victor 1420 multilabel counter (PerkinElmer, Rodgau, Germany).

### Cell Migration

Influence of HSP90 inhibitors on cell migration was determined using the wound healing assay (ibidi GmbH, Martinsried, Germany). Specifically, NCI-H295R (3.7 × 10^4^ per chamber) and MUC-1 (1 × 10^4^ per chamber) cells were seeded and incubated overnight. At the following day, cells were treated with 0.005 v/v charcoal stripped serum media containing 0.01 v/v penicillin/streptomycin and HSP90 inhibitors (luminespib, ganetespib, novobiocin, and silibinin). In addition, mitomycin C (10 μM, Roth GmbH & Co. KG, Karlsruhe, Germany) was added to inhibit proliferation. Images were taken at different time points (0, 9, 24, and 48 h) and cell-free area was quantified using ImageJ Software.

### Immunoblotting

NCI-H295R and MUC-1 cell proteins were extracted in RIPA buffer (50 mM Tris pH8.0, 150 mM NaCl, 0.01 v/v NP-40 (Sigma-Aldrich), 0.005 v/v sodium deoxycholate (Sigma-Aldrich), and 0.001 v/v SDS (Roth) supplemented with complete protease inhibitor cocktail (Roche) and phosphatase inhibitor cocktail (Sigma-Aldrich). The homogenized lysate was centrifuged at 16,000 g for 15 min and protein concentration was quantified by Bradford protein assay (Bio-Rad, Hercules, Ca, USA) following the manufacturer's recommendations. Twenty microgram of protein solution was re-suspended in Roti-Load (Roth) loading buffer and boiled for 5 min at 95°C. Sample separation was done in a 0.1 v/v polyacrylamide gel and then transferred onto a nitrocellulose membrane (Merck-Millipore, Kenilworth, United States). After blocking in 0.05 v/v non-fat milk [diluted in TBST (137 mM NaCl, 2.7 nM KCl (Sigma-Aldrich), 19 mM TrisHCl, 0.1 % Tween®20 (Sigma-Aldrich)] at RT for 1 h, membranes were incubated overnight with primary antibodies, following three washes for 5 min. at RT and incubation for 1 h at RT with corresponding HRP-conjugated secondary anti-mouse (7074S, 1:2000, Cell Signaling) or anti-rabbit (7076S, 1:2000, Cell Signaling), diluted in 0.05 v/v non-fat dry milk. After three additional washes, proteins of interest were detected using enhanced chemiluminescence (ECL) solution (Merck-Millipore) in a Molecular Imager® ChemiDoc™ XRS+ with Image Lab™ Software (Bio-Rad). The list of primary antibodies used in this study is available on Table 1.

**Table 1:** List of antibodies used for immunoblot analysis.

**Target**	**Size (KDa)**	**Dilution**	**Cat.No**	**Manufacturer**
AKT	60	1:2000	4,691	Cell signaling
p-Ser473 AKT	60	1:2000	9,271	Cell signaling
β-ACTIN	43	1:5000	MAB1501	Merck-millipore
ERK 1/2	42,44	1:4000	9,102	Cell signaling
p-ERK 1/2	42, 44	1:4000	9,101	Cell signaling
GR	91, 94	1:2000	12,041	Cell signaling
HSP90α	90	1:1000	PA3-013	Thermo fisher
HSP90β	90	1:1000	PA3-012	Thermo fisher
MEK 1/2	45	1:1000	9,122	Cell signaling
p-Ser217/221 MEK 1/2	45	1:1000	9,121	Cell signaling
c-Raf	65 to 75	1:1000	9,422	Cell signaling
p-Ser338-c-Raf	74	1:1000	9,427	Cell signaling
RAS	20	1:1000	3,965	Cell signaling
RSK	90	1:1000	9,355	Cell signaling
p-Ser380 RSK	90	1:1000	11,989	Cell signaling
mTOR	289	1:1000	2,972	Cell signaling
p-Ser2448 TOR	289	1:1000	2,971	Cell signaling

*β-ACTIN was used as protein loading control*.

### Cortisol Measurement

NCI-H295R (8 × 10^5^ cells per well) were seeded in 6 well plates and left overnight to recover. Treatment for 6 and 24 h with HSP90 inhibitors was performed at the following day, supernatants were collected at each time point and cortisol measurement was conducted by Liaison, Diasorin, Sallugia Italy. Cell viability was quantified by MTT assay as described above.

### Statistical Analysis

Semi-quantitative HSP90 immunostaining was correlated with clinical parameters of patients from group 1 using the Spearman's rank correlation, Wilcoxon rank-sum test and Kruskal-Wallis-Test. In addition, for patients from group 2, HSP90 immunostaining was evaluated for differences in patient survival determined using the Kaplan-Meier log-rank test. Progression-free survival (PFS) was defined as time elapsed from primary resection of ACC to the first recurrence of disease while overall survival (OS) was defined as time elapsed from primary resection of ACC to disease-related death or last follow-up visit. Multivariate survival analysis was performed using Cox proportional hazards regression models. All calculations were performed using R version 3.4.3 with the survival package^3^.

For *in vitro* experiments, results were evaluated with GraphPad Prism (Houston, TX, USA) and illustrated as mean ± SD. Statistical significance was determined using analysis of variance (ANOVA) or Kruskal-Wallis-test, followed by Bonferroni's or Dunnett correction for multiple comparisons. Synergy scores were calculated with web application^4^ using the ZIP method (50).

Statistical significance was defined as *P* < 0.05 and denoted as stars for the highest used concentration of HSP90 inhibitor (^*^P < 0.05; ^**^P < 0.01; ^***^P < 0.001; ^****^P < 0.0001) in all figures, if not stated otherwise.

## Results

### HSP90 Immunostaining as a Prognostic Marker of ACC

**Figure 1:**
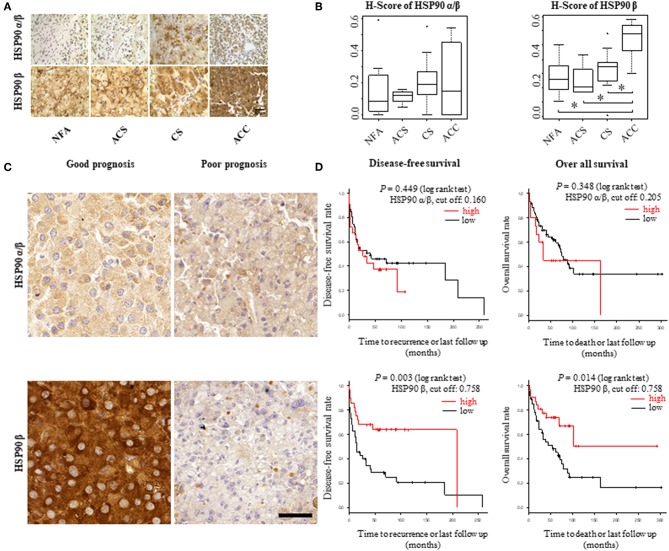
HSP90α/β and HSP90β staining in adrenocortical adenomas (NFA, non-functional adenoma, *n* = 8; ACS, adenoma with autonomous cortisol secretion, *n* = 4; CS, adenoma with overt Cushing syndrome, *n* = 14) and adrenocortical carcinoma (ACC, *n* = 6) with representative sections **(A)** and semi-quantitative H-scores **(B)**. Representative images of HSP90α/β and HSP90β immunohistochemistry from an ACC patient with good prognosis without recurrence and one with poor prognosis with disease related death, respectively **(C)**. Kaplan-Meier plots for disease-free and overall survival **(D)** in relation to HSP90α/β and HSP90β cytoplasmic intensity above (high: red line) or below (low: black line) the cut off. Log rank test was used to statistically compare the curves and *P*-values are provided (*P < 0.05).

To explore the role of HSP90α/β and β as potential biomarkers in adrenal tumors, their expression was evaluated by immunohistochemistry in FFPE samples from patient group 1, which included 32 patients with non-functional adenomas (NFA), adenomas associated with autonomous cortisol secretion (ACS) and overt Cushing's syndrome (CS) and ACC, respectively (Supplemental Table 1A). Immunoreactivity was quantified as H-scores. Expression levels for HSP90β were higher in ACC samples (*n* = 6) in comparison to adenomas (CS, *n* = 14; SCS, *n* = 4; NFA, *n* = 8) (Figures 1A,B). In contrast, no clear differences among the groups were evident for HSP90α/β staining intensities.

For further analysis within ACC samples, cytoplasmic intensities of HSP90α/β and β were quantified in a cohort of 80 ACC patients (patient group 2; Supplemental Table 1B) using digital image analysis. Following correlation with clinical characteristics, lower intensity of HSP90β was associated with hormone-producing ACCs (Supplemental Figure 1). On the other hand, there were no significant correlations between cytoplasmic intensities of HSP90α/β and β and clinical parameters such as age at diagnosis, Weiss score and Ki67 index (Supplemental Table 2). In addition, Cox proportional hazards regression revealed a significant relationship between low cytoplasmic intensities of HSP90β and disease-free survival and overall survival, while no such correlation was observed between cytoplasmic intensities of HSP90α/β (Figures 1C,D). As expected, clinical factors such as age at diagnosis, cortisol production and ENSAT stage were significantly correlated with disease-free survival (Supplemental Table 3A) and overall survival (Supplemental Table 3B). Similarly, Weiss scores were associated with overall survival (Supplemental Table 3B). Interestingly, multivariate analysis using clinical factors revealed that low cytoplasmic HSP90β staining intensity remained an independent factor for disease free survival (Table 2A), while only cortisol production and ENSAT stage were significant for overall survival (Table 2B). In addition, multivariate analysis including Ki67 index and cytoplasmic intensities of HSP90β showed that both factors were independent regarding disease free survival (Supplemental Table 4A) and overall survival (Supplemental Table 4B).

**Table 2:** Multivariate analysis using Cox proportional hazard ratio model for disease free survival **(A)** and overall survival **(B)** (HR, hazard ratio; CI, confidence interval).

**A**				
**Disease free survival covariates**	**Cut off (category)**	**HR**	**95%CI**	***P-*value**
Age at diagnosis, years	≥61 (vs. <61)	1.507	0.648–3.502	0.341
Cortisol production	Yes (vs. No)	2.248	1.139–4.435	0.020
ENSAT stage	IV (vs. I, II, and III)	4.560	1.604–12.96	0.004
Cytoplasmic intensity of HSP90β	<0.760(vs. ≥0.760)	2.574	1.183–5.598	0.017
**B**				
**Overall survival covariates**	**Cut off (category)**	**HR**	**95%CI**	***P-*****value**
Age at diagnosis, years	≥60 (vs. <60)	1.694	0.814–3.524	0.159
Cortisol production	Yes (vs. No)	2.584	1.270–5.259	0.009
ENSAT stage	IV (vs. I, II, and III)	7.676	3.314–17.78	<0.001
Cytoplasmic intensity of Hsp90β	<0.760(vs. ≥0.760)	1.672	0.761–3.671	0.200

### Abundance and Localization of HSP90α/β and HSP90β Isoforms in ACC Xenografts and ACC Cell Lines

**Figure 2:**
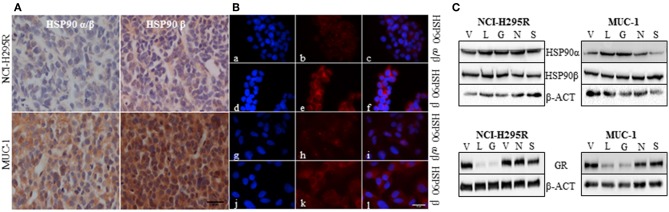
Representative pictures of the abundance of HSP90α/β and HSP90β in NCI-H295R and MUC-1 xenografts **(A)**. Immunofluorescence staining illustrating the localization of HSP90α/β (a, g: DAPI; b,h: HSP90α/β; c,i: merged) and HSP90β (d,j: DAPI; e,k: HSP90β; f,l: merged) in NCI-H295R (a–f) and MUC-1 (g–l) cells **(B)**. Expression of HSP90α, HSP90β and glucocorticoid receptor (GR) was demonstrated by Western Blot analysis. NCI-H295R and MUC-1 cells were treated with HSP90 inhibitors for 48 h vehicle (V): 1:1000 DMSO; luminespib (L): 0.2 μM; ganetespib (G): 0.2 μM; novobiocin (N): 100 μM; silibinin (S): 40 μM **(C)**. Scale bars represent 25 μm.

Immunohistochemical analysis of HSP90α/β and HSP90β in NCI-H295R and MUC-1 xenograft tissues demonstrated a higher abundance of both isoforms in MUC-1 xenografts compared to those of NCI-H295R (Figure 2A). With respect to subcellular distribution, immunofluorescence staining of NCI-H295R and MUC-1 cells demonstrated both a nuclear and cytoplasmic localization of HSP90α/β, while HSP90β was found to be localized primarily in the cytoplasm (Figure 2B). Expression of HSP90α, HSP90β and the glucocorticoid receptor (GR) were further quantified by Western Blot analysis. In both cell lines, treatment with either C- or N-terminal HSP90 inhibitors had no effect on the expression of HSP90α and HSP90β. In contrast, luminespib and ganetespib treatment resulted in a decrease of GR expression in both cell lines (Figure 2C), as also described for other cell lines (51).

### N-terminal HSP90 Inhibitors Display Anti-tumor Efficacy as Single Agents Against ACC Cells

**Figure 3:**
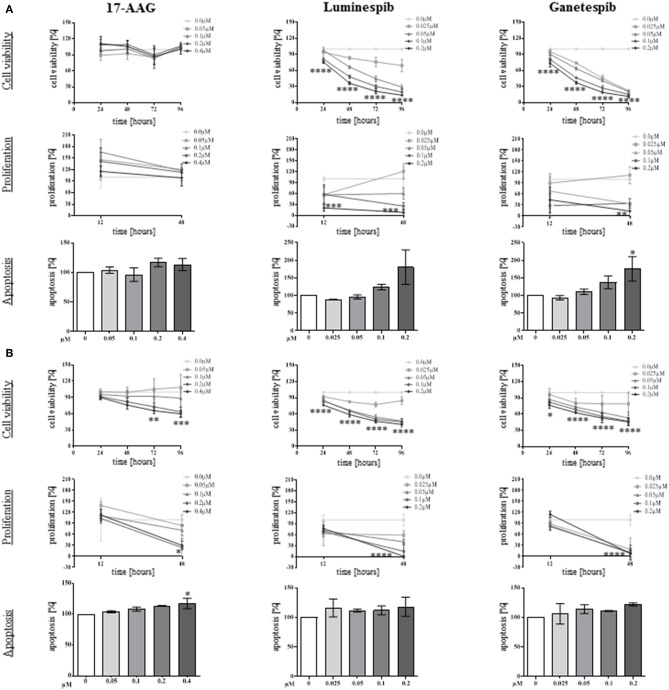
Anti-tumor properties of N-terminal HSP90 inhibitors investigated in NCI-H295R **(A)** and MUC-1 **(B)** cells. Using different drug concentrations and following different time points, efficacy on cell viability (upper panel), cell proliferation (middle panel), and apoptosis (lower panel) were quantified. Analysis were done in triplicates and statistical analysis was performed using analysis of variance (*P < 0.05; **P < 0.01; ***P < 0.001; ****P < 0.0001).

To determine the anti-tumor effectiveness of N-terminal and C-terminal HSP90 inhibitors, cell viability was investigated in single and combined treatment protocols. Thereby, NCI-H295R and MUC-1 cell viability was significantly affected in a time and dosage dependent manner by single use of luminespib and ganetespib while 17-AAG demonstrated significant effects only in MUC-1 cells (Figures 3A,B).

To further provide insights whether the effects of HSP90 inhibitors on cellular viability are accompanied by inhibition of cell growth, proliferation assays for both cell lines were included in the analysis. In fact, luminespib and ganetespib induced strong anti-proliferative effects in both cell lines after 48 h of treatment, but only MUC-1 cells responded to 17-AAG as well (Figures 3A,B).

A further increase of apoptosis was observed by single use of luminespib and ganetespib in NCI-H295R cells after 24 h of treatment (Figure 3A). In variance with NCI-H295R cells, no significant increase of apoptosis was identified upon luminespib and ganetespib treatment in MUC-1 cells (Figure 3B).

**Figure 4:**
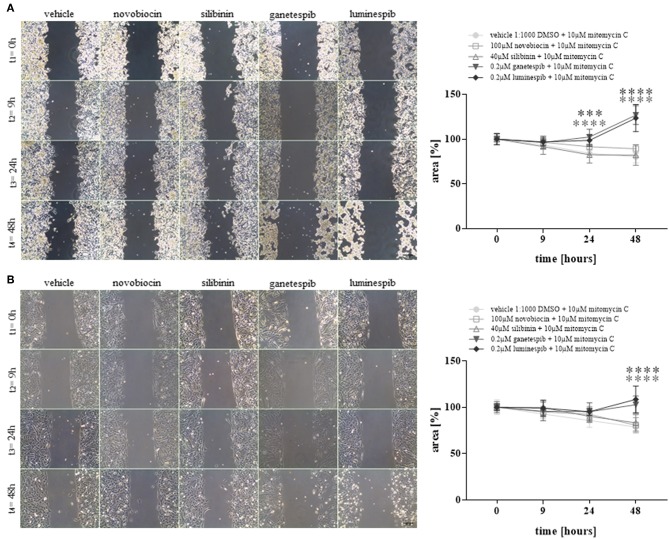
Cell migration analysis at different time points and with different HSP90 inhibitors in NCI-H295R **(A)** and MUC-1 **(B)** cells. Treatment was performed in presence of mitomycin C to avoid cell proliferation. Representative pictures of three independent experiments are shown and statistical analysis was performed using analysis of variance. Scale bars represent 200 μm (***P < 0.001; ****P < 0.0001).

Additionally, cell migration assays demonstrated strong significant effects upon luminespib and ganetespib in NCI-H295R (Figure 4A) and MUC-1 (Figure 4B) cells in comparison to vehicle treatment. In contrast, C-terminal HSP90 inhibitors were ineffective on cell migration compared to vehicle treated cells (Figures 4A,B).

In summary, neither silibinin (S) nor novobiocin (N) illustrated any effects toward cell viability, cell proliferation, apoptosis or cell migration in any cell lines following the dosages and timing of the current experimental conditions (Figure 4 and Supplemental Figure 2). In contrast, the N-terminal inhibitors luminespib and ganetespib were effective in both cell lines, with more pronounced effects on the induction of apoptosis in NCI-H295R cells and more relevant inhibition of proliferation in MUC-1 cells, respectively.

**Figure 5:**
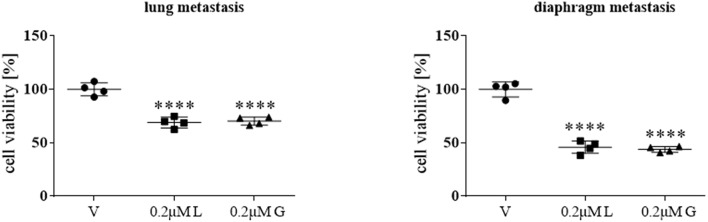
Anti-tumor effects of 0.2 μM luminespib (L) and 0.2 μM ganetespib (G) vs. vehicle (V) were analyzed in ACC primary culture cells of the same patient. The illustrations demonstrate the effects on cell viability in lung and diaphragm metastasis. Measurements were performed with one ACC primary culture in quadruplicates and statistical analysis was done using analysis of variance (****P < 0.0001).

Following the initial screening and in-depth characterization of a range of HSP90 inhibitors, primary cultures from two distinct metastatic sites (lung and diaphragm) of a patient with stage IV ACC were treated for 72 h with 0.2 μM luminespib and ganetespib, respectively. HSP90 inhibitors revealed significant treatment effects by luminespib and ganetespib (Figure 5).

### Determination of Combinatory Effects of N-terminal HSP90 Inhibitors

**Figure 6:**
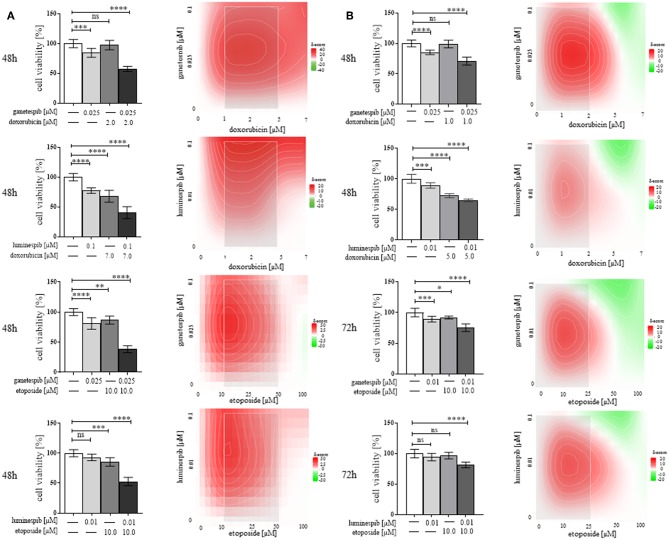
Demonstration of efficacy of N-terminal HSP90 inhibitors (luminespib and ganetespib) in combination with compounds of the clinically used EDP-M scheme on cell viability in NCI-H295R **(A)** and MUC-1 **(B)** cells. Effects are based on three independent experiments with bar charts and heatmaps using analysis of variance, where red color indicates combined effects and green color provides indication of non-combined effects (*P < 0.05; **P < 0.01; ***P < 0.001; ****P < 0.0001).

Following the success of single treatment with luminespib and ganetespib, we further explored whether additive or synergistic effects would be in concomitance with the standard EDP-M scheme. In fact, relevant dosage and time dependent synergistic effects were evident particularly following co-treatment with doxorubicin and etoposide, respectively (Figures 6A,B). In contrast, cisplatin and mitotane demonstrated weak additional effects when used in combination with the different HSP90 inhibitors (ganetespib, luminespib, silibinin; novobiocin) in both cell lines (data not shown).

### Molecular Effects of HSP Inhibitors in Adrenocortical Cancer Cell Lines

**Figure 7:**
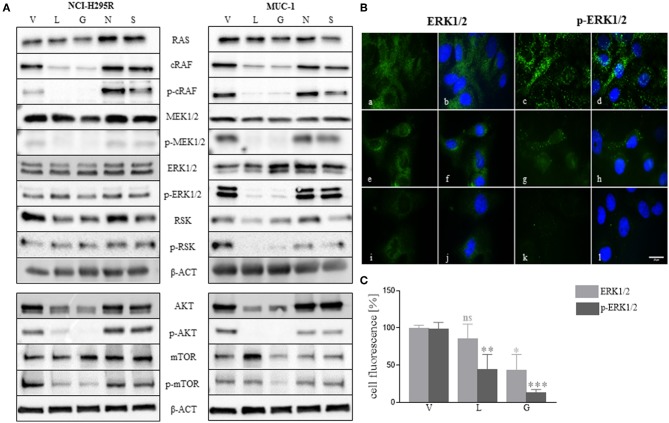
Effects of HSP90 inhibition on ERK1/2 and AKT pathways were investigated in NCI-H295R and MUC-1 cells after 48 h treatment with HSP90 inhibitors luminespib (L): 0.2 μM, ganetespib (G): 0.2 μM, novobiocin (N): 100 μM, and silibinin (S) 40 μM, vehicle (V): 1/1000 DMSO. Representative blots of two to three independently experiments are shown **(A)**. Representative immunofluorescence pictures of ERK1/2 and p-ERK1/2 demonstrated a significant reduction of ERK1/2 activation upon treatment with luminespib and ganetespib **(B,C)**. V: 1:1000 DMSO 48 h (a-d); L: 0.2 μM 48 h (e–h); G: 0.2 μM 48 h (i–l). Analysis of three pictures from each staining and treatment was performed. Scale bars represent 25 μm (*P < 0.05; **P < 0.01; ***P < 0.001).

As demonstrated above, NCI-H295R cells were more sensitive to single and combined treatment than MUC-1 cells (Figures 3, 6), and showed different response on cell proliferation and apoptosis. To further elucidate those observations, we investigated the effects of HSP inhibition on intracellular pathways. Specifically, analysis of the ERK1/2 (extracellular-regulated kinase 1/2) pathway indicated a decrease of p-cRAF and p-MEK1/2 forms upon luminespib and ganetespib treatment in both cell lines. Interestingly, a decrease of p-ERK1/2 and further downstream targets of ERK1/2 pathway was observed upon luminespib and ganetespib treatment only in MUC-1 cells. Immunofluorescence staining also proved significant decrease of p-ERK1/2 in luminespib and ganetespib treated MUC-1 cells (Figures 7B,C). In contrast, C-terminal HSP90 inhibitors were ineffective on ERK1/2 pathway in NCI-H295R and MUC-1 cells (Figure 7A). Additionally, we investigated the AKT-pathway. A decrease of p-AKT and p-mTOR was demonstrated by the used N-terminal HSP90 inhibitors, luminespib and ganetespib, in both adrenocortical carcinoma cell lines (Figure 7A).

In conclusion, functional activity on cell lines as assessed by cell viability is mirrored to some extent by molecular profiles in pathways known to have impact in adrenocortical tumorigenesis.

### Modulation of Steroidogenesis Upon HSP90 Inhibitor Treatment

**Figure 8:**
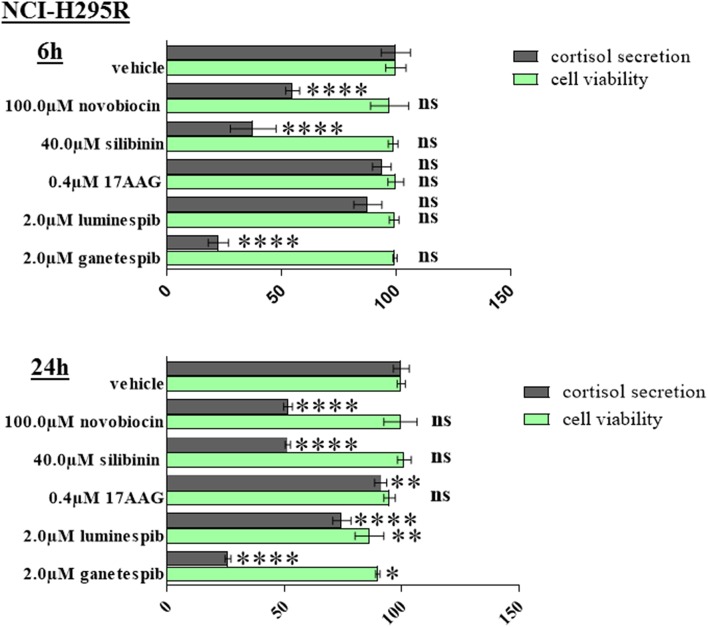
NCI-H295R demonstrated significant reduction of cortisol secretion after 6 and 24 h treatment with HSP90 inhibitors. Analysis were done in triplicates and statistical analysis was performed using analysis of variance (*P < 0.05; **P < 0.01; ****P < 0.0001).

Significant reduction of cortisol secretion can be induced after novobiocin, silibinin, and ganetespib treatment already at time points when no relevant effects on cellular viability is observed. Provided the low steroidogenic capacity of MUC-1 cells we have restricted the experiments on NCI-H295R cells (Figure 8).

## Discussion

Herein we provide evidence that expression levels of isoforms of HSP90 bear prognostic value in ACC patients and that targeting HSP90 function has therapeutic potential resulting in consistent anti-tumor effects in different cell systems of adrenocortical cancer origin.

Specifically, we demonstrate that HSP90β expression is significantly higher in ACC in comparison to benign adrenocortical adenomas independently of their endocrine activity, while HSP90α/β is more evenly distributed among these tumor entities. These results confirm the general observation that HSP90 is produced at a higher level in cancer cells (52). It is interesting to note, however, that within the group of ACCs, the more malignant phenotype was associated with lower HSP90β expression, which translates into a decrease in recurrence-free and overall survival. This association was maintained even in multivariate analysis providing indirect evidence for a functional impact of this feature on tumor biology. In contrast, HSP90α/β expression has no relevant prognostic impact.

Over the last decade, it has become increasingly clear that ACC—similar to other tumor entities—comprise a heterogeneous group of tumors that differ in their genetic and molecular set-up, hormonal capacity and malignant behavior. A number of immunohistochemical markers have been identified that are associated with prognostic value. However, in many instances it remains uncertain whether the difference in expression levels is in causal relationship with the clinical characteristics or caused by other molecular events. As for the lower expression of HSP90β it remains to be shown in functional studies whether this finding is cause or consequence of malignant clinical characteristics.

Considering the limited treatment opportunities for ACC patients, there is a continuous need to explore new therapeutic modalities in a therapeutic setting. HSP inhibition has recently been postulated as a promising avenue for targeting of ACC (53, 54). Our current data provide *in vitro* evidence for a potential use of HSP inhibitors in the treatment of ACC patients. Specifically, we show that later generation N-terminal HSP90 inhibitors, such as luminespib and ganetespib, display strong anti-tumor activity with effects on cell viability, cell proliferation, cellular migration, and to a lesser extent on apoptosis. For potential clinical applications, these substances have been described to possess improved bioavailability and to be afflicted with less side effects than geldanamycin derivatives, such as 17-AAG (55). Nevertheless, also 17-AAG provided some treatment responses in our *in vitro* system. It is interesting to note that the cell line with lower expression of HSP90β–NCI-H295R—overall showed higher responsiveness toward HSP90 inhibition. While these data are too preliminary to suggest a marker of treatment response, they inform that even low HSP90β expression levels are sufficient to allow for a therapeutic impact of HSP90 inhibitors.

In current clinical practice, ACC patients not amendable to surgery are treated with a combination of cytostatic drugs including etoposide, doxorubicin, and cisplatin in combination with the adrenolytic substance mitotane (56, 57). Recent evidence has indicated that the combination of the HSP90 inhibitors BIIB021 and CCT18159 with mitotane had limited additional value (53). In contrast, following our current investigation, we can provide a rational for a combined treatment of luminespib or ganetespib with doxorubicin or etoposide as these combinations demonstrated additive or synergistic effects, especially in NCI-H295R cells. As the response rates of current chemotherapies are generally low, combinations with further active substances could be a strategy to improve therapeutic efficacy. At the same time, drug related side effects, which are mostly dosage dependent, could be overcome by combination schemes with lower dosages of the single agents.

Tumor heterogeneity is a well-appreciated basis of treatment failure, because different tumor clones can bear diverse functional characteristics including primary drug resistance. Initiation of therapies increases selective pressure and may drive evolution of a clone with pre-existing or newly acquired functional properties that overcome therapeutic response. In the present study, we have made usage of two different cells lines and primary cultures from two different metastatic sites of one ACC patient. In all instances, *in vitro* treatment with N-terminal HSP90 inhibitors resulted in strong effects on cell viability. Thereby, these findings—together with the widespread expression of HSP90 in ACC samples—provide first indication for a likely more general treatment response of ACC patients toward HSP90 inhibitors. The MUC-1 cell line, which had been derived from a metastasis of a patient pre-treated with several cycles of EDP (48) was found to be less responsive to HSP90 inhibition. While these subtle differences in treatment response are to be expected, it is interesting to note, that the cell lines also differed with a more pronounced inhibition of cell proliferation for MUC-1 cells while NCI-H295R cells were more prone to undergo treatment related apoptosis.

A number of client proteins of HSP90, including glucocorticoid receptor and kinases play an important role in cancer signaling pathway (58). Similar to recent findings in pituitary cells (39), treatment of adrenocortical cancer cell lines with N-terminal HSP90 inhibitors decreased their glucocorticoid receptor expression and additionally we observed a reduction of cortisol secretion in NCI-H295R cells upon C-terminal and N-terminal HSP90 inhibitor treatment at time points without relevant effects on cell viability. We could further demonstrate that, in line with their relevant anti-tumor activity, N-terminal HSP90 inhibitors induce changes in molecular pathways involved in tumorigenesis. Among them, AKT and ERK1/2 pathways have recently attracted attention as potential therapeutic targets for ACC. Moreover, HSP90 inhibitors also affect AKT and ERK1/2 pathways (59–62). Interestingly, we can demonstrate that luminespib and ganetespib treatment leads to inhibition of phosphorylated AKT and mTOR in both investigated cell lines. Similarly, analysis of ERK1/2 pathway by western blot further indicated a decrease of p-cRAF and p-MEK1/2 both in NCI-H295R and MUC-1 cells. However, only in MUC-1 cells we observed a reduction of p-ERK1/2 and p-RSK upon luminespib and ganetespib treatment. It is prudent to speculate that these differential molecular treatment responses could be related with the observed differences in treatment response between both cell lines.

We appreciate the limitation of the current study as the functional analysis are based on two cell lines with further supporting data from two individual ACC metastases from one patient. Based on the recently reported progress in the establishment of further human adrenocortical cells lines (63), there is hope that future preclinical studies in the field can rely on additional models that open the possibilities to study for further individualized approaches.

In summary, new generation N-terminal HSP90 inhibitors hold promise as agents with anti-tumor properties against adrenocortical cancer cells. Furthermore, low expression of HSP90β could be used as prognostic marker for ACC patients. Following subsequent studies in appropriate preclinical models could pave the way toward the introduction of N-terminal HSP90 inhibitors in the treatment of patients with ACC.

## Data Availability

All datasets generated for this study are included in the manuscript and/or the Supplementary Files.

## Ethics Statement

All patients had provided written informed consent and the study was approved by ethics committees at all participating institutions (Medizinische Fakultät der Universität München, Maastrich University, Hôpital Cochin Paris, University of Florence, and Universität Würzburg).

## Author Contributions

CS, DC, LF-S, LP-R, and JM-G performed the majority of the *in vitro* experiments. MMu calculated the survival curves. SN and JM executed *in vitro* analyses on primary cultures. EK provided the tissue microarray. AF and AW supported staining and quantification of immunohistochemistry. HH, JB, MMa, MF, and MR provided tumor samples and clinical annotations, while GS, CH, and FB planned and overlooked the experiments. All authors actively participated in writing of the manuscript.

### Conflict of Interest Statement

The authors declare that the research was conducted in the absence of any commercial or financial relationships that could be construed as a potential conflict of interest.

## Footnotes

^1^https://clinicaltrials.gov/ct2/results?term=AUY-922&age_v=&gndr=&type=&rslt=With&phase=1&phase=2&Search=Apply; https://clinicaltrials.gov/ct2/results?term=ganetespib&age_v=&gndr=&type=&rslt=With&Search=Apply

^2^www.ensat.org

^3^https://github.com/therneau/survival

^4^https://synergyfinder.fimm.fi/
