# Supplementary material for: Heat Shock Protein 90 as a Prognostic Marker and Therapeutic Target for Adrenocortical Carcinoma
Source: Front Endocrinol (Lausanne). 2019 Jul 19;10:487. doi: 10.3389/fendo.2019.00487 (PMC6658895; doi:10.3389/fendo.2019.00487)
Supplement: Supplementary file 1 [file Data_Sheet_1.ZIP › Supplemental Material_table 1.docx]

**Supplemental Material**

**Supplemental Tables**

**Supplemental Table 1**: Clinical characteristics of 32 patients with adrenal tumor (patient group 1, **A**) and 80 patients with ACC (patient group 2, **B**). Data are expressed as mean ± SD; n, (%); or median (IQR).

**A**

| Age at diagnosis (years) | 49.0 | ± | 17.6 |
| --- | --- | --- | --- |
| Tumor Size (cm) | 4.0 |  | (3.0 - 5.8) |
| Sex | 5: male |  | 28: female |
| Tumor type (n, %) |  |  |  |
| Nonfunctional adenomas | 8 |  | 25% |
| Cortisol-secreting adenoma | 18 |  | 56% |
| - autonomous cortisol secretion | 4 |  |  |
| - overt Cushing’s syndrome | 14 |  |  |
| Adrenocortical carcinoma | 6 |  | 19% |

**B**

| Age at diagnosis (years) | 50.0 | ± | 17.6 |
| --- | --- | --- | --- |
| Tumor Size (cm) | 10.3 |  | (7.5 - 15.1) |
| Sex | 33: male |  | 47: female |
| Weiss score | 6 |  | (6 - 7) |
| Ki67 index | 14 |  | (5 - 30) |
|  |  |  |  |
| Hormonal activity (n, %) |  |  |  |
| Nonfunctional | 33 |  | 41% |
| Cortisol | 34 |  | 43% |
| Aldosterone | 1 |  | 1% |
| Sex steroids | 23 |  | 29% |
|  |  |  |  |
| Metastasis (n, %) | 52 |  |  |
| Disease related death (n, %) | 40 |  |  |
|  |  |  |  |
| ENSAT stage (n, %) |  |  |  |
| stage I | 6 |  | 8% |
| stage II | 36 |  | 45% |
| stage III | 16 |  | 20% |
| stage IV | 16 |  | 20% |
