# Supplementary material for: Heat Shock Protein 90 as a Prognostic Marker and Therapeutic Target for Adrenocortical Carcinoma
Source: Front Endocrinol (Lausanne). 2019 Jul 19;10:487. doi: 10.3389/fendo.2019.00487 (PMC6658895; doi:10.3389/fendo.2019.00487)
Supplement: Supplementary file 1 [file Data_Sheet_1.ZIP › Supplemental Material_table 2.docx]

|  | cytoplasmic intensity | | | |
| --- | --- | --- | --- | --- |
|  | HSP90α/β | | HSP90β | |
|  | r_s_ | *P*-value | r_s_ | *P*-value |
| Age at diagnosis | -0.141 | 0.212 | -0.072 | 0.523 |
| Weiss score | 0.057 | 0.654 | -0.040 | 0.753 |
| Ki67 index | 0.090 | 0.457 | 0.139 | 0.249 |

**Supplemental Table 2**: Correlations between clinical parameters and cytoplasmic intensities of HSP90α/β and β.
