# Supplementary material for: Heat Shock Protein 90 as a Prognostic Marker and Therapeutic Target for Adrenocortical Carcinoma
Source: Front Endocrinol (Lausanne). 2019 Jul 19;10:487. doi: 10.3389/fendo.2019.00487 (PMC6658895; doi:10.3389/fendo.2019.00487)
Supplement: Supplementary file 1 [file Data_Sheet_1.ZIP › Supplemental Material_table 3.docx]

**Supplemental Table 3**: Relationships between clinical characteristics and disease-free survival (A) and overall survival (B), analyzed with univariate Cox proportional hazard ratio model within ACC patients (HR, hazard ratio; CI, confidence interval).

**A**

| Factors | Cut off (Category) | HR | 95%CI | *P* value |
| --- | --- | --- | --- | --- |
| Sex | M (vs F) | 1.373 | 0.751-2.510 | 0.301 |
| Age at diagnosis, years | >=61 (vs <61) | 2.713 | 1.358-5.420 | 0.003 |
| Weiss score | 9 (vs <9) | 3.513 | 0.772-15.98 | 0.083 |
| Ki67 index | >=6 (vs <6) | 5.151 | 1.978-13.41 | <0.001 |
| Hormonal activity | Yes (vs No) | 1.839 | 0.947-3.570 | 0.068 |
| Cortisol production | Yes (vs No) | 2.853 | 1.512-5.384 | <0.001 |
| Sex steroids production | Yes (vs No) | 0.958 | 0.487-1.887 | 0.902 |
| ENSAT staging | IV (vs I,II and III) | 6.205 | 2.468-15.60 | <0.001 |

**B**

| Factors | Cut off (Category) | HR | 95%CI | *P* value |
| --- | --- | --- | --- | --- |
| Sex | M (vs F) | 1.164 | 0.623-2.172 | 0.634 |
| Age at diagnosis | >=60 (vs <60) | 2.721 | 1.410-5.25 | 0.002 |
| Weiss score | 9 (vs <9) | 8.509 | 2.592-27.94 | <0.001 |
| Ki67 index | >=12 (vs <12) | 5.109 | 2.266-11.52 | <0.001 |
| Hormonal activity | Yes (vs No) | 1.874 | 0.961-3.654 | 0.061 |
| Cortisol production | Yes (vs No) | 2.877 | 1.515-5.463 | <0.001 |
| Sex steroids production | Yes (vs No) | 0.894 | 0.445-1.797 | 0.753 |
| ENSAT staging | IV (vs I,II and III) | 8.176 | 3.692-18.11 | <0.001 |
