# Supplementary material for: Heat Shock Protein 90 as a Prognostic Marker and Therapeutic Target for Adrenocortical Carcinoma
Source: Front Endocrinol (Lausanne). 2019 Jul 19;10:487. doi: 10.3389/fendo.2019.00487 (PMC6658895; doi:10.3389/fendo.2019.00487)
Supplement: Supplementary file 1 [file Data_Sheet_1.ZIP › Supplemental Material_table 4.docx]

**Supplemental Table 4:** Multivariate analysis using Cox proportional hazard ratio model for disease free survival (A) and overall survival (B) (HR, hazard ratio; CI, confidence interval).

**A**

| **Disease free survival**  Covariates | Cut off (category) | HR | 95%CI | *P-*value |
| --- | --- | --- | --- | --- |
| Ki67 index | >=6 (vs <6) | 5.052 | 1.932-13.21 | <0.001 |
| Cytoplasmic intensity of HSP90β | <0.760(vs >=0.760) | 2.356 | 1.104-5.029 | 0.027 |

**B**

| **Overall survival**  Covariates | Cut off (category) | HR | 95%CI | *P-*value |
| --- | --- | --- | --- | --- |
| Ki67 index | >=12 (vs <12) | 5.559 | 2.411-12.82 | <0.001 |
| Cytoplasmic intensity of Hsp90β | <0.760(vs >=0.760) | 2.294 | 1.057-4.976 | 0.036 |
