# Supplementary figures and images for: Heat Shock Protein 90 as a Prognostic Marker and Therapeutic Target for Adrenocortical Carcinoma
Source: Front Endocrinol (Lausanne). 2019 Jul 19;10:487. doi: 10.3389/fendo.2019.00487 (PMC6658895; doi:10.3389/fendo.2019.00487)

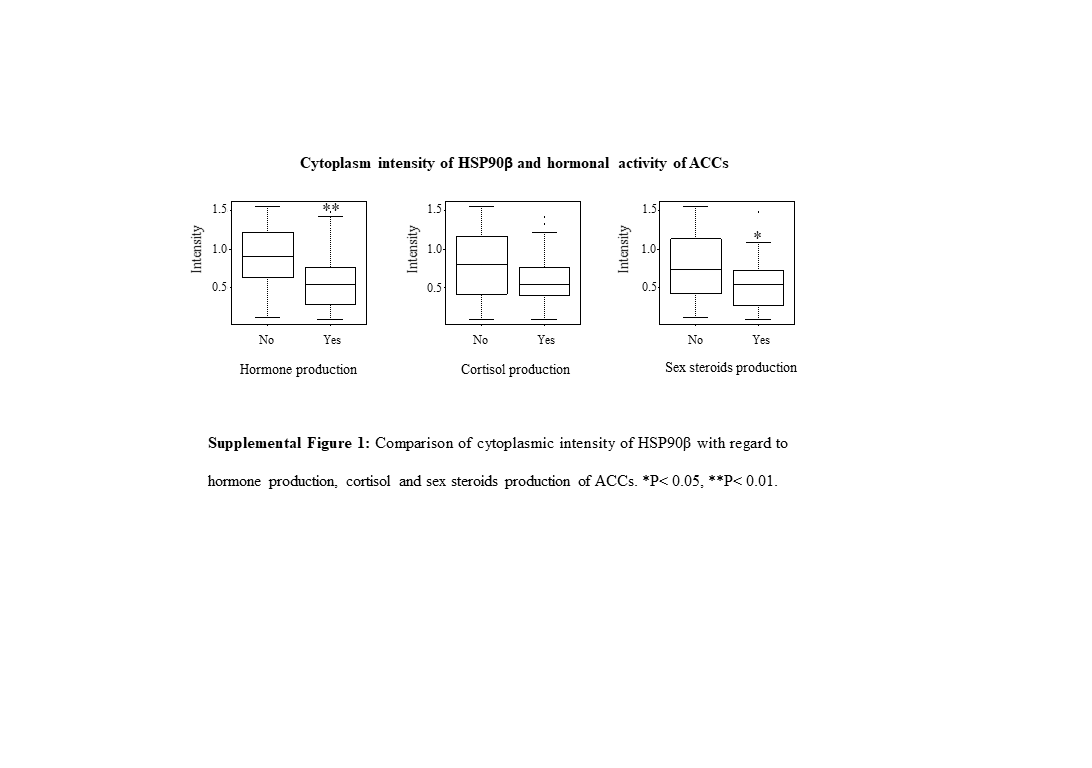

Supplement: Supplementary file 1 [file Data_Sheet_1.ZIP › Supplemental Figure_1.tif]

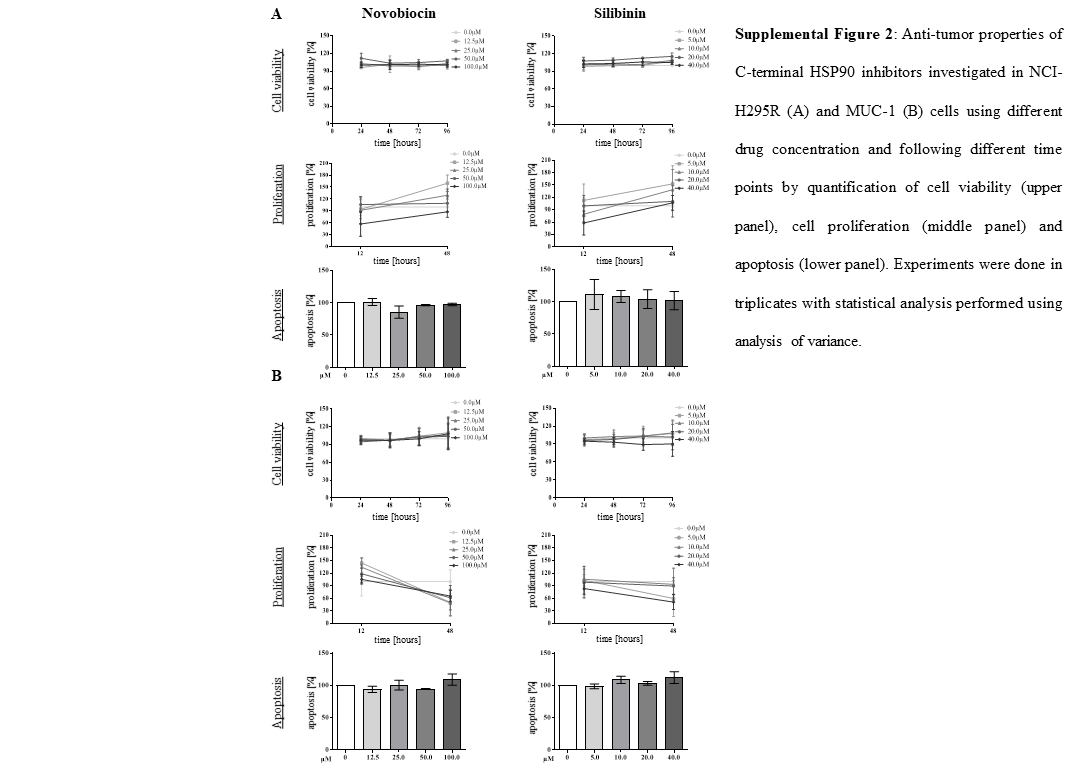

Supplement: Supplementary file 1 [file Data_Sheet_1.ZIP › Supplemental Figure_2.tif]
